# Evaluation of Microhardness of Mineral Trioxide Aggregate After Immediate Placement of Different Coronal Restorations: An In Vitro Study

**Published:** 2018-03

**Authors:** Maryam Kazemipoor, Niloofar Azizi, Farnaz Farahat

**Affiliations:** 1 Assistant Professor, Department of Endodontics, School of Dentistry, Shahid Sadoughi University of Medical Sciences, Yazd, Iran; 2 Dentist, Private Practice, Yazd, Iran; 3 Assistant Professor, Department of Operative Dentistry, School of Dentistry, Shahid Sadoughi University of Medical Sciences, Yazd, Iran

**Keywords:** Dental Restoration, Mineral Trioxide Aggregate, Composite Resins, Glass Ionomer Cements

## Abstract

**Objectives:**

The purpose of this research was to evaluate the effect of immediate placement of different restorative materials in comparison with a temporary restoration on the surface microhardness of mineral trioxide aggregate (MTA).

**Materials and Methods:**

Access cavities were prepared in 40 extracted human molars, and a 3-mm layer of MTA was placed in the pulp chamber. The samples were divided into eight groups (n=5). Ten minutes after the MTA placement, two groups were restored with Zonalin temporary restoration, while the other six groups were restored with glass-ionomer cement (GIC), resin-modified glass-ionomer (RMGI), or resin-based composite. In each group, the Vickers microhardness (VMH) of MTA was determined after 7 and 21 days. Data were entered into SPSS 17 software program and were analyzed by two-way analysis of variance (ANOVA). The significance level was set at 5%.

**Results:**

The type of restorative materials had a statistically significant effect on the microhardness of MTA (P=0.002). However, the microhardness of MTA was neither significantly influenced by the timing of final restoration (P=0.246) nor by the time-material interaction (P=0.116).

**Conclusions:**

Based on the results of the present study and by considering the limitations of laboratory studies, it is recommended to postpone the placement of final restorations until the underlying MTA is completely set. Otherwise, in the clinical conditions in which early covering of MTA is recommended, sufficient moist-curing and hydration should be guaranteed by selecting a restorative material with the lowest hydrophilic interaction energy.

## INTRODUCTION

Mineral trioxide aggregate (MTA) was first introduced in 1993 as a root-end filling material [1]. This cement is composed of a hydrophilic powder which reacts with water and sets through hydration process [2]. Nowadays, MTA is also used for pulp capping, pulpotomy, pulpectomy, apical sealing in open-apex teeth, repairing of perforations, and filling root canals [1]. The advantages of MTA include biocompatibility, radiopacity slightly greater than that of dentin, low solubility, high alkalinity (pH=12.5), and antibacterial and antifungal effects [2]. In addition, MTA is an active biological material for osteoblasts, and it stimulates interleukin production because of the alkaline pH and release of calcium ions [3]. However, the main disadvantages of MTA are difficult manipulation, tooth discoloration, long setting time, and solubility during the setting period. These limitations may affect MTA properties during the setting process [2,4, 5]. The average setting time of MTA is 165 minutes, which is longer than the setting time of many of the existing restorative materials [5]. After mixing the hydrophilic powder with water, a colloidal gel is made which forms a solid barrier after 3 to 4 hours, while the complete setting reaction of MTA may take about 23 days [2,5, 6]. Since the early set time of MTA is about 3 to 4 hours, an additional session may be needed for the placement of final restoration [2]. However, immediate placement of the final coronal restoration is very important for the promotion of the coronal seal and treatment prognosis. Immediate coronal seal with a permanent restoration leads to less microleakage and increased treatment success [7,8]. Common materials for coronal restoration include resin-based composite, glass-ionomer cement (GIC), resin-modified GI (RMGI) and amalgam. The effect of immediate coronal restoration on the physical properties of MTA has been evaluated in few studies [1,7–9]. Also, the effect of the time of coronal restoration on the surface microhardness of MTA has been assessed only in a survey by Tsujimoto et al [1]. Therefore, the aim of the present study was to investigate the effect of immediate placement of resin composite, GIC, and RMGI in comparison with a temporary filling material on the surface microhardness of MTA. The null hypothesis was that the microhardness of MTA would not be affected by the type of filling materials.

## MATERIALS AND METHODS

### Preparation of samples:

In this in-vitro experimental study, 40 extracted human molars with mature apices and without any root resorption, canal calcification, endodontic treatment, or coronal restoration were selected. After extraction, the teeth were cleaned and stored in 0.5% Chloramine-T solution at 4°C before the preparation of the samples. An access cavity was prepared in each tooth, and the pulp chamber was rinsed with 5.25% sodium hypochlorite (NaClO) solution followed by a rinse with normal saline. The root canals were filled with normal saline to the level of canal orifice.

According to the manufacturer’s instruction, the powder and liquid of the MTA (Angelus, Londrina, PR, Brazil) were mixed with the standard ratio, and a 3-mm layer of the mixture was placed in the pulp chamber. A moistened cotton pellet was placed on the MTA surface, and the teeth were randomly divided into eight groups (n=5) according to the type of coronal restoration:

Control groups (groups 1 and 2): A 2-mm layer of a temporary filling material (Zonalin, Golchai, Tehran, Iran) was placed on the wet cotton pellet. Next, the teeth were incubated at 37°C and 100% relative humidity for 7 (group 1) and 21 days (group 2).
RMGI groups (groups 3 and 4): The cotton pellet was removed after 10 minutes. The powder and liquid of the RMGI (GC Fuji II LC, Tokyo, Japan) were mixed with the standard ratio according to the manufacturer’s instruction, and a 2-mm layer of the mixture was placed over the MTA. The RMGI was cured for 20 seconds by using a light-emitting diode (LED) curing light (Demi^TM^ Plus, Kerr Dental Co., California, USA) with the intensity of 900 mW/cm^2^. The samples were then incubated at 37°C and 100% relative humidity for 7 (group 3) and 21 days (group 4).
GIC groups (groups 5 and 6): The cotton pellet was removed after 10 minutes. The powder and liquid of the GIC (GC Fuji XI, GC Co., Tokyo, Japan) were mixed with the standard ratio according to the manufacturer’s instruction, and a 2-mm layer of the mixture was placed over the MTA. After the completion of the initial setting reaction of the GIC (10 minutes), the samples were incubated at 37°C and 100% relative humidity for 7 (group 5) and 21 days (group 6).
Resin composite groups (groups 7 and 8): The cotton pellet was removed after 10 minutes. The self-etch primer and bonding agent of the Clearfil™ SE Bond (Kuraray Medical Inc., Okayama, Japan) were applied according to the manufacturer’s instruction. Afterwards, a 2-mm thickness of the flowable composite resin (Clearfil™ Majesty Flow, A3 shade, Kuraray Medical Inc., Okayama, Japan) was placed over the MTA and was cured for 40 seconds by using the LED curing light. The teeth were then incubated at 37°C and 100% relative humidity for 7 (group 7) and 21 days (group 8).

### Vickers microhardness (VMH) testing:

The samples were mounted in a custom-made mold by using a self-curing acrylic resin (Asia ChemiTeb Co, Tehran, Iran). The teeth were sectioned longitudinally by using a low-speed saw and were polished with silicon carbide papers (300 to 1200 grit).

In each sample, VMH testing was performed by using a VMH tester (Micromet® 5114, Buehler Ltd., Lake Bluff, IL, USA) at three points and at a 200-μm distance from the MTA-filling material interface by applying a 500 gram-force (gf) load with the dwell time of 10 seconds. The angle between the opposite faces of the diamond indenter was 136 degrees. The diameter of the indentation was measured at each point, and the mean of three points was calculated as the Vickers hardness number (VHN) of each sample in Kg/mm^2^.

### Statistical analysis:

Data were analyzed by using SPSS version 17 software program (IBM Co., Chicago, IL, USA). The effects of the type and time of placement of the filling material on the microhardness of MTA were measured by two-way analysis of variance (ANOVA). The significance level was set at 5%.

## RESULTS

The results of the present study are summarized in Table 1. Based on two-way ANOVA, there were significant differences in the microhardness of MTA with respect to the type of restorative materials (P=0.002). However, the microhardness of MTA was not significantly influenced by the time of restoration (P=0.246) or by the time-material interaction (P=0.116). The mean VHN in the groups treated with Zonalin was significantly higher than that of the samples treated with either GIC (P=0.01) or resin composite (P<0.001).

**Table 1.** Mean and standard deviation (SD) of the Vickers hardness number (VHN; Kg/mm^2^) of mineral trioxide aggregate (MTA) at 7 and 21 days

**Time**	**7 days**	**21 days**

**Filling material**		
Zonalin	29.16±14.00	47.02±19.34
RMGI	29.71±7.45	28.20±4.49
GIC	22.58±11.08	27.11±7.24
Flowable resin composite	20.25±9.32	15.53±5.00

RMGI=Resin-Modified Glass-Ionomer, GIC= Glass-Ionomer Cement

The VHN also showed significant statistical differences between the RMGI and resin composite groups (P=0.029).

## DISCUSSION

The MTA powder is a refined Portland cement that is extensively used in endodontics. This bioceramic has two main characteristics that differentiate it from other available restorative materials: biocompatibility and a superior sealing ability [10].

Based on the results of a meta-analysis, MTA is the most biocompatible substance when compared with Super-EBA^®^ (2-ethoxybenzoic acid), IRM^®^ (Intermediate Restorative Material), and amalgam [11]. During the initial setting, the biocompatibility of a restorative material placed in close contact with vital tissues is more important for improving the treatment success rate [12]. However, after the completion of the setting process, the sealing ability of a restorative material, to prevent the leakage of irritants from the root canal system into adjacent tissues, becomes more prominent for clinical success [10].

The sealing ability of MTA depends on physical and chemical properties of the material after the finalization of the setting process [13]. Studies have demonstrated that after MTA is placed over pulpal tissue, a hydroxyapatite layer forms over the material in contact with tissue fluids and also at the interface of the restorative material and dentinal walls, which leads to the construction of a biologic seal [14,15].

In the clinical settings, especially when MTA is applied to the coronal part of the tooth, the physical properties of the material such as surface microhardness also play an important role in achieving an ideal seal [1]. The occlusal loads during mastication may lead to displacement of the restorative material and disruption of the physical seal. Since microhardness of a material is directly related to the setting reaction, any factor that interferes with the MTA setting could affect the microhardness of MTA and could hamper the physical seal [5,8].

Although the long setting time of MTA may result in a two-session application of this material, immediate placement of a final restoration could promote the coronal seal [1]. Moreover, based on the results of clinical studies, the prognosis of direct pulp capping with MTA does not depend on the timing of the final restoration [16].

In case of immediate coronal restoration, clinical manipulations including the condensation pressure, etching, rinsing, and priming could affect the setting of MTA [1]. Since the effect of the immediate placement of a coronal restoration on the physical properties of the underlying MTA has not been studied extensively, the present survey was conducted to investigate the effect of the immediate placement of light-curing RMGI, self-curing GIC, and resin composite in comparison with Zonalin temporary restoration.

Based on the results of the present study, the null hypothesis was rejected, and there were significant differences between the groups in terms of the microhardness of MTA after the use of different restorative materials; however, the time of final restoration and the time-material interaction did not significantly change the microhardness of MTA. In the groups evaluated after 7 days, the highest and lowest mean VHNs were recorded for Zonalin/RMGI and resin composite, respectively. In the groups evaluated after 21 days, the Zonalin groups showed the highest, while the resin composite groups showed the lowest mean VHNs. During the examination time, the VHN increased in the Zonalin and GIC groups, while it decreased in the resin composite and RMGI groups. The ultimate microhardness of MTA was similar in the RMGI and GIC groups.

Yesilyurt et al [8] measured the shear bond strength of conventional GICs to MTA after 45 minutes and after 72 hours of the placement of a coronal restoration and concluded that GICs can be placed over MTA in a single-visit procedure. Nandini et al [7] also demonstrated that a single-visit coronal restoration with GIC did not affect the setting of MTA or formation of calcium salt at the MTA-restoration interface.

Tsujimoto et al [1] investigated the proper time to restore resin composites over MTA. A flowable resin composite was placed over MTA at 10 minutes, 1 day, and 7 days after the MTA placement. Afterwards, the distance between the two materials and the 28-day microhardness of MTA were measured. The VHNs in the groups evaluated after one day were significantly lower than those in the 10-minute, 7-day and control groups. Although the type of resin composite in the present study is the same as the type used in the study by Tsujimoto et al [1], the differences in the obtained data are the result of different times of the placement of the final restoration. In the present study, the final restoration was placed 10 minutes after the MTA placement in all the samples, and the VHNs were measured after 7 and 21 days; however, in the study by Tsujimoto et al [1], coronal restorations were placed after 10 minutes, 1 day, and 7 days, and the microhardness was recorded after 28 days. Also, the duration of contact between the moist cotton pellet and MTA was different in the two studies. The condition of the two studies was almost similar only with regard to the groups evaluated after 10 minutes and with regard to VHN assessments after 21–28 days. Different types of tested MTA (Angelus in the present study vs. ProRoot MTA in the study by Tsujimoto et al [1]) with different physical properties [17], different study samples (natural teeth in the present study vs. silicone tubes in the study by Tsujimoto et al [1]), and differences in the Vickers indenter loads (500gf with the dwell time of 10 seconds in the present study vs. 50gf with the 5-second dwell time in the study by Tsujimoto et al [1]) may be the reasons for the observed discrepancies in the obtained data.

Patil et al [18] evaluated the GIC-MTA interface and the effect of the time of restoration on this interface. They reported that the cohesive separation in MTA was more prominent when the GIC was condensed 45 minutes after the MTA placement in comparison with the immediately condensed GIC. In addition, the conventional GIC showed a better adhesion to MTA in comparison with the light-curing GIC [18]. These results are in agreement with the findings of the present study indicating the superiority of GIC to other types of final restoration. However, the study designs of the two surveys are different. Eid et al [2] investigated the effect of the MTA setting conditions and RMGIC placement time on the microhardness of the MTA interface. Twenty-four hours after the GIC placement, the VHNs revealed a significant increase in hardness with an increased temporization time but not with a change in moisture conditions. Based on the results of the cited study, GICs can be placed over a freshly mixed MTA with minimal effect on the microhardness of MTA [2]. Since the temporization, moisture contact time, study model, and hardness indenter load in the cited study are different from those in the present study, we observed differences between the two surveys with respect to the importance of the effect of temporization on the VHN results.

Based on the outcomes of the current study, the application of the self-etching resin composite significantly reduced the microhardness of MTA. The initial setting of MTA is not completed 10 minutes after the MTA placement, and a 10-minute moist-curing of MTA may not be sufficient for the completion of the setting process. Therefore, the early covering of MTA with a resin composite is not recommended.

Many factors could influence the setting reaction and microhardness of a bioactive material like MTA [5,19]. The condensation pressure, amount of entrapped air in the mixture, the material’s thickness, humidity, temperature, pH values of the environment, application of ethylenediaminetetraacetic acid (EDTA), and acid-etching may interfere with the setting process and may influence the final microhardness of MTA [20,21].

More condensation, less material thickness, the presence of a chelating agent, and acid-etching might adversely affect the microhardness of MTA [5]. An evaluation of the effect of the environment on the microhardness of MTA indicated that during the hydration phase, needle-like and dominant cubic crystals are formed within MTA [4]. The formation and growth of these needle-like crystals between cubic crystals are directly correlated with the final microhardness of the material [4]. An acidic environment prevents the formation of the needle-like crystals and leads to a decreased microhardness [4].

According to Lee et al [4], the physiological environment affects the crystal formation in MTA through the pH and the presence of ions. The microstructures of the samples stored in normal saline or distilled water having a pH of 7 are different as they show larger crystals and supplementary laminate formation on the outer surface of the cubic crystals in normal saline (pH=7). Therefore, in the present study, the root canals were filled with normal saline with a pH of 7.

Since the moisture cannot effectively diffuse through thick layers of MTA, a bilateral moisture exposure has been recommended for the completion of the setting process [22]. Gancedo-Caravia and Garcia-Barbero [23] reported that keeping MTA in contact with a moistened cotton pellet for 21 days significantly affects the push-out strength. In this regard, the final VHNs of MTA in the present study were measured after 21 days.

There are contradictory results with regard to the impact of the moist-curing time on the final microhardness of MTA [1,8,24,25]. In the present study, the microhardness values in the RMGI groups were higher than those in the GIC and resin composite groups. This is probably because of the water sorption of GIC and the hydrophilic nature of the primer in the resin composite package that could interfere with the hydration and crystallization of the underlying MTA during the setting process [26].

## CONCLUSION

In the clinical conditions in which early covering of MTA is recommended, sufficient moist-curing and hydration should be guaranteed by selecting a restorative material with the lowest hydrophilic interaction energy. Otherwise, it is recommended to postpone the placement of the final restoration to after the completion of the MTA setting process.
